# High Detection Rates of Enteropathogens in Asymptomatic Children Attending Day Care

**DOI:** 10.1371/journal.pone.0089496

**Published:** 2014-02-24

**Authors:** Remko Enserink, Rianne Scholts, Patricia Bruijning-Verhagen, Erwin Duizer, Harry Vennema, Richard de Boer, Titia Kortbeek, Jeroen Roelfsema, Henriette Smit, Mirjam Kooistra-Smid, Wilfrid van Pelt

**Affiliations:** 1 Center for Infectious Disease Control (Epidemiology and Surveillance Unit), National Institute for Public Health and the Environment (RIVM), Bilthoven, The Netherlands; 2 Center for Infectious Disease Control (Laboratory for Infectious Diseases and Perinatal Screening), National Institute for Public Health and the Environment (RIVM), Bilthoven, The Netherlands; 3 Julius Center for Health Sciences and Primary Care, University Medical Center Utrecht, Utrecht, The Netherlands; 4 Laboratory for Infectious Diseases, Department of Research and Development, Groningen, The Netherlands; 5 Department of Medical Microbiology, University of Groningen, University Medical Center Groningen, Groningen, The Netherlands; University of North Carolina School of Medicine, United States of America

## Abstract

**Background:**

Gastroenteritis morbidity is high among children under the age of four, especially amongst those who attend day care.

**Objective:**

To determine the prevalence of a range of enteropathogens in the intestinal flora of children attending day care and to relate their occurrence with characteristics of the sampled child and the sampling season.

**Methods:**

We performed three years of enteropathogen surveillance in a network of 29 child day care centers in the Netherlands. The centers were instructed to take one fecal sample from ten randomly chosen children each month, regardless of gastrointestinal symptoms at time of sampling. All samples were analyzed for the molecular detection of 16 enteropathogenic bacteria, parasites and viruses by real-time multiplex PCR.

**Results:**

Enteropathogens were detected in 78.0% of the 5197 fecal samples. Of the total, 95.4% of samples were obtained from children who had no gastroenteritis symptoms at time of sampling. Bacterial enteropathogens were detected most often (most prevalent EPEC, 19.9%), followed by parasitic enteropathogens (most prevalent: *D. fragilis*, 22.1%) and viral enteropathogens (most prevalent: norovirus, 9.5%). 4.6% of samples related to children that experienced symptoms of gastroenteritis at time of sampling. Only rotavirus and norovirus were significantly associated with gastroenteritis among day care attendees.

**Conclusions:**

Our study indicates that asymptomatic infections with enteropathogens in day care attendees are not a rare event and that gastroenteritis caused by infections with these enteropathogens is only one expression of their presence.

## Introduction

Gastroenteritis is a major cause of morbidity in children aged 0 through 3 years worldwide [1]. Even in industrialized countries such as the Netherlands, with high standards of sanitation and water quality, gastroenteritis morbidity is high among children under four years of age [2], [3], especially amongst those who attend day care. For example, in Dutch child day care centers (DCCs), the risk of developing gastroenteritis was found to be three times higher than national estimates for this age group in the general population [4]. DCCs in the Netherlands provide care for half of the approximately 0.7 million Dutch children aged 0–4 years. Given the assumed impact of day care-associated gastroenteritis on the attending child and the outbreak potential of the DCC setting, public health authorities support DCCs in their duty to control infectious diseases. To provide such support, these authorities need up-to-date and accurate estimates on the seasonal prevalence of a broad range of enteropathogens. Such estimates provide the baseline for studies of disease burden, cost of illness, risk factors, and intervention and help to assess the impact of gastroenteritis trends in the day care setting. Results from other studies help in this regard, although most pertain to well-known etiologic agents of mostly viral origin [5]–[7] during gastroenteritis outbreak investigations [8], [9], or to investigations in the setting of hospitals or general practice [2], [10]. In addition, these studies are often characterized by selective stool testing of symptomatic individuals, while a far larger number of gastrointestinal infections appear sporadically and possibly asymptomatically.

Using three years of surveillance data from a national surveillance network of DCCs, the objectives of this study were to (1); provide estimates of the prevalence of a range of enteropathogens of bacterial, parasitic and viral origin in the intestinal flora of children attending day care and to (2); relate the prevalence of these enteropathogens with the child’s age and gastroenteritis status at time of sampling as well as the season and the year of sampling.

## Methods

### Ethics Statement

This study was performed as part of a larger, ongoing, national day care-based surveillance network on the occurrence of and risk factors for infectious diseases in Dutch child day care [11]. This network has a prospective cohort design, following day care centers rather than individual children or staff members over time. The Dutch Central Committee on Research involving Human Subjects in Utrecht, The Netherlands, gave permission to conduct this study (protocol number: 09–196/C). Given that limited subject-identifiable data were generated and the surveillance activities implied no risk or burden for any individuals, the committee judged that no specific ethical permission was required for institutional or individual consent. Although not required, parents or guardians of children attending participating DCCs were informed by letter of the purpose and design of the study and an information form was attached that parents could return if they did not want to let their children participate in the study.

### Setting

Recruitment among 3913 Dutch DCCs took place from November 2009 to April 2010 using a database that included all Dutch DCCs operating in the Netherlands at that time. DCCs received an invitation to complete an attached questionnaire and subsequently to participate in enteropathogen surveillance activities amongst their child attendees. They were asked to participate for at least one year. Additional recruitment was performed in March 2011 and March 2012 to ensure additional inflow in the day care cohort. For a detailed description regarding the objectives, design and creation of the KIzSS network, we refer to our study-design article [11].

Of the approached DCCs, 2612 DCCs (67%) did not respond at all and 356 DCCs (9%) indicated that they lacked time and/or interest to participate in either the survey among DCCs or the surveillance network as reported previously. Among the 945 DCCs that participated in the national questionnaire survey, 18 centers (2%) started with enteropathogen surveillance activities in March 2009. In total, 29 centers (3%) participated in surveillance activities during the study period. The representativeness of the DCC surveillance network was thoroughly assessed by statistical and principal component analysis in a study published earlier this year [4]. This study confirmed that participating DCCs were representative of the Dutch DCC population with regard to socioeconomic status, urbanization degree, size, staff-to-child ratio, and group structure.

### Fecal Sample Collection

DCCs were instructed to take one fecal sample from 10 randomly chosen children each month (one sample per child), regardless of whether or not these children experienced gastrointestinal symptoms at time of sampling and regardless of the size of the DCC. Per sample taken, DCCs were instructed to document sampling identifiers, including the sampling date and the child’s age, gender, and presence of gastrointestinal symptoms. Such presence was defined as at least one episode of loose or liquid stools and/or vomiting during the three days prior to sampling. The probability of two children having identical sampling identifiers was considered small as each center had been informed that repeated sampling of the same child within the same month was not allowed. Samples with identical limited identifiers were therefore considered to be taken from one and the same child. Samples taken within the same months from the same child were excluded from further analysis. DCCs stored fecal samples locally at 4°C before sending the samples to the Research & Development department at the Laboratory for Infectious Diseases (LvI) in Groningen, the Netherlands.

### Sample Preparation for Total Nucleic Acid Extraction

The LvI prepared samples for total nucleic acid (TNA) extraction (MediaProducts BV., Groningen, The Netherlands) as described previously [12]. From each sample, a fecal suspension was prepared according to a pre-extraction protocol for fecal samples (bioMérieux, release 1) and consequently stored at −20°C to await TNA extraction. From the same fecal sample, a selenite-enriched broth was inoculated and incubated for approximately 24 h at 35°C. A part (1 ml) of the selenite enrichment broth was stored at −20°C to await TNA extraction. The remainder of the selenite broth and feces suspension was respectively stored at room temperature and 4°C until further culture, depending on the real-time multiplex PCR (mPCR) results. After mPCR results and/or culture, the feces samples and TNA were stored at −80°C until they were sent to the National Institute for Public Health and the Environment (RIVM) to be stored for future reference.

### Total Nucleic Acid Extraction

After sample preparation, the LvI performed TNA extraction from both the fecal suspension and O/N selenite broth using an automated NucliSens easyMAG (bioMérieux). Briefly, 100 µl of fecal suspension and 50 µl O/N selenite broth were mixed together and used as input. Phocine herpesvirus 1 (PhHV) and Equine arteritisvirus (EAV) were co-purified and served as internal controls (IC). TNAs were eluted in 110 µl of elution buffer. Every extraction run included a negative and a positive extraction control. The latter consisted of a pooled fecal suspension that was spiked with target organisms that could be detected with a real-time multiplex PCR (mPCR) for *Salmonella enterica* (*S*.*enterica*), *Campylobacter jejuni* (*C. jejuni*), shigatoxin producing *Escherichia coli* (STEC), and *Shigella* spp./EIEC. Also, a positive DNA control was used that could detect the target organisms *Clostridium difficile* (*C*. *difficile*), enteroaggregative *Escherichia coli* (EAEC), typical and atypical enteropathogenic *Escherichia coli* (EPEC) and *Yersinia enterocolitica* (*Y*. *enterocolitica*).

### Molecular Detection of Enteropathogenic Bacteria, Viruses and Parasites

Molecular detection of enteropathogens was performed either by the LvI (bacterial enteropathogens) or by the RIVM (viral and parasitic enteropathogens). The LvI performed molecular detection of bacterial gastrointestinal pathogens by targeting *S. enterica* [12], *C. jejuni* [12], *C. difficile* [13], *Y. enterocolitica* [2], *Shigella* spp./Enteroinvasive *E. coli* (EIEC) [12], Shigatoxin-producing *E. coli* (STEC) [12], Enteroaggregative *E. coli* (EAEC) [2], and Enteropathogenic *E. coli* (EPEC) [2] in four internally controlled quantitative real-time multiplex polymerase chain reactions (mPCRs). The primers and probes, and the set-up of the qPCR reactions are available on request. mPCR positive samples for *S. enterica*, *Shigella* spp./EIEC, STEC, and EPEC were cultured on selective growth media from the stored stool specimen or selenite enrichment broth. A sample was considered positive for the presence of an enteropathogen if the enteropathogen was detected by mPCR. The procedures for culturing *S. enterica* and *Shigella* spp. have been previously described [13]. Briefly, culturing for STEC and EPEC was carried out on Sorbitol McConkey agar (48 h at 35°C). Suspect *E. coli* colonies (sorbitol and non-sorbitol fermenting) were sub-cultured for genotyping and serotyping of STEC and EPEC. For EPEC, the suspect *E. coli* colonies were tested by PCR for the presence of the locus of enterocyte effacement region (escV) and the EPEC adherence factor plasmid (bfpA). Attempts to isolate STEC and typical EPEC were made up to a maximum of five colonies per stool sample. PCR positive results for *C. difficile* were subtyped for the detection of the tcdC Δ1171bp deletion, associated with *C. difficile* PCR ribotype 027 as described previously [13]. After detection, the LvI sent all remaining fecal material (unpreserved fecal samples and fecal suspensions) and TNA eluates to the RIVM for detection of viral and parasitic enteropathogens and further molecular typing.

The RIVM laboratory performed molecular detection of parasitic gastrointestinal pathogens by targeting *Giardia lamblia* (*G. lamblia*), *Cryptosporidium hominis* and *parvum* (*C. hominis* and *C. parvum*), and *Dientamoeba fragilis* (*D. fragilis*) in one internally controlled qPCR. A standard program of 10 s at 95°C, 20 s at 58°C and 20 s at 72°C for 45 cycles was used with the FastStart kit from Roche. Primer and probe sequences have been described by Verweij et al. [14], [15]. We used a 6Fam- and BHQ1-labelled probe for G. *lamblia*, Texas Red- and BHQ2-labelled probes for *Cryptosporidium* spp.; a Vic and BHQ1 labelled probe for *D. fragilis,* and a Cy5 and BHQ2 labelled probe for phocid herpesvirus, the internal control. We typed all positive *G. lamblia* samples using the Tpi gene to reveal the assemblage types (A and B). Similarly, we typed positive Cryptosporidium samples using the GP60 gene to differentiate between *C. hominis* and *C. parvum*, suggestive for antroponotic or zoönotic transmission routes respectively.

Finally, the RIVM laboratory performed molecular detection of viral gastrointestinal pathogens by targeting norovirus, adenovirus (enteropathogenic types 30 and 40/41), sapovirus, astrovirus and rotavirus in three parallel qPCRs by applying a two-step method. In the first cDNA step, multiple target sequences belonging to the gastrointestinal viruses mentioned were amplified using random primers. In the second step, three parallel PCR assays were conducted to detect (1) noroviruses of genogroup I, II and II.4 and rotavirus group A; (2) adenovirus, sapovirus and astrovirus; and (3) norovirus GI and EAV Detected viruses were genotyped by partial genome sequencing of the capsid gene (norovirus, adenovirus, sapoviruses, astrovirus), or by PCR-based genotyping protocols (rotavirus) [16]. All remaining fecal materials were stored at −80 degrees in a central biobank at RIVM for future reference.

### Statistical Analyses

The crude prevalence was the number of fecal samples positive for a specific enteropathogen divided by the total number of fecal samples analyzed during the study period. Samples derived from the same child during the same month at the same DCC were excluded from further analysis, as were samples for which outcome or exposure information was missing.

Firstly, we fitted pathogen-specific multilevel mixed-effects (MME) logistic regression models to estimate the odds ratios for the associations between the presence of the enteropathogen under study (outcome) and the child and seasonal covariates (exposure variables) of age, season, year, and gastrointestinal symptoms at time of sampling. These models included two random-effects, one at the level of the DCC and one at the level of the individual child. These random effects accounted for any dependency in the data due to the clustering at the level of the DCC and the possibility of repeated sampling of the same child over time. Secondly, using the fitted MME logistic regression models, we estimated the prevalence of each enteropathogen in the feces of asymptomatic children aged of 0–2 years old during the winter season of 2011/2012. We provided adjusted prevalence estimates for asymptomatic children rather than for symptomatic children as symptomatic children were likely to be underrepresented in our study because of day care absence due to illness. Other than the GE status of the child, this fecal sample profile was chosen as the probability of detecting an enteropathogen in such a sample would be high. All covariates in the model were either binary or categorical. The age of the child was classified as 0–2 years or 3–4 years. The seasons were classified as spring (March 21 to June 21); summer (June 21 to September 21); autumn (September 21 to December 21) and winter (December 21 to March 21). Finally, the years were defined as the periods between March 21, 2010 to March 21, 2011; March 21, 2011 to March 21, 2012 and March 21, 2012 to March 21, 2013. We analyzed all data using the statistical software package STATA/SE 12 for Windows.

## Results

Twenty-nine DCCs participated during the study period. The average participating center cared for a median of 35 children [5–95% percentiles: 10–70]. Individual DCCs sent in a median of 9 samples per month (2.5–97.5 percentile: 4–14 samples) for a median period of 11 months (2.5–97.5 percentile: 1–31 months). Altogether, they sent in 5590 fecal samples during the study period. Of these fecal samples, we excluded 46 samples from children aged more than 3 years of age and 347 samples from children sampled more than once in the same month in the same DCC, leaving 5197 samples.

Table 1 provides estimates of the enteropathogen prevalence and their associations with the child’s age and gastroenteritis status as well as the season and the year of sampling. An enteropathogen was detected in the majority of the samples analyzed (4053/5197 samples or 78.0%). 1803 fecal samples (34.7%) contained mixed infections of two (25.0%), three (7.9%), four (1.5%), five (0.2%) or six enteropathogens (one sample). Approximately 95% of fecal samples originated from children who showed no signs or symptoms of gastroenteritis at time of sampling. Enteropathogenic bacteria were detected most often (2323/5197 samples or 44.7%), followed by parasites (1403/5197 samples or 27.0%) and viruses (1149/5197 samples or 22.1%). Of the enteropathogenic bacteria, EPEC was most prevalent (19.9%), followed by *C. difficile* (16.5%), EAEC (5.3%), STEC (1.9%), *C. jejuni* (0.5%), *S. enterica* (0.3%), *Shigella spp*./EIEC (0.1%) and *Y. enterocolitica* (0.1%). Of the parasites, *D. fragilis* was the most often detected enteropathogen (22.1%), followed by *G. lamblia* (4.2%) and *C. hominis* and *C. parvum* (0.8%). Of the viruses, norovirus was most frequently detected (9.5%), followed by sapovirus (3.9%), rotavirus (3.3%), astrovirus (2.8%), and finally adenovirus (2.7%). A third of fecal samples contained mixed infections of 2 (1064 samples, 20.5%), 3 (673, 12.9%), 4 (151, 2.9%), 5 (25, 0.5%), or 6 enteropathogens (4, 0.1%).

**Table 1:** Prevalence of enteropathogens in the feces of children attending day care from March 2010 through March 2013 in the Netherlands.

N = 5197 fecal samples	N samples	Prevalence(%, crude*)	Prevalence(%, estimated**)	Age categoryOR*** [95% CI]Ref: 0–2 years	SeasonOR [95% CI]Ref: winter	Study yearOR [95% CI]Ref: ‘11–‘12	GastroenteritisOR [95% CI]Ref: no complaints
**BACTERIAL PATHOGEN**	2323	44.7 [43.1–46.3]	58.4 [52.7–63.9]	**0.7 [0.6–0.7]**	**2.0 [1.7–2.5]**	1.0 [0.9–1.0]	1.0 [0.9–1.2]
Enteropathogenic *E. coli*	1035	19.9 [18.8–21.0]	31.6 [26.0–32.4]	1.0 [0.8–1.2]	**5.0 [3.3–5.5]**	1.1 [1.0–1.2]	1.2 [0.9–1.5]
*Clostridium difficile*	857	16.5 [15.5–17.5]	25.0 [18.7–32.4]	**0.1 [0.1–0.2]**	0.9 [0.7–1.1]	0.9 [0.8–1.0]	0.9 [0.7–1.2]
Enteroaggregative *E. coli*	276	5.3 [4.7–5.7]	3.6 [1.9–6.5]	0.9 [0.6–1.3]	**10.0 [3.3–10.5]**	0.8 [0.6–1.0]	0.7 [0.4–1.4]
Shigatoxin-producing *E. coli*	101	1.9 [1.6–2.3]	4.1 [2.1–7.9]	1.3 [0.7–2.3]	1.3 [0.8–2.0]	1.1 [0.8–1.6]	0.9 [0.3–2.3]
*Campylobacter jejuni*	15	0.5 [0.3–0.7]****	–	–	–	–	–
*Salmonella enterica* *	28	0.3 [0.1–0.4]****	–	–	–	–	–
*Shigella* spp.	6	0.1 [0.0–0.2]****	–	–	–	–	–
*Yersinia enterocolitica*	5	0.1 [0.0–0.2]****	–	–	–	–	–
**PARASITIC PATHOGEN**	1403	27.0 [25.7–28.3]	18.2 [13.3–24.6]	**2.3 [2.0–2.7]**	1.1[0.9–1.3]	1.0 [0.9–1.1]	1.1 [0.9–1.3]
*Dientamoeba fragilis*	1151	22.1 [21–23.3]	16.9 [12.1–23.1]	**2.4 [2.1–2.9]**	1.1 [1.0–1.7]	1.0 [0.9–1.1]	1.0 [0.8–1.2]
*Giardia lamblia*	217	4.2 [3.6–4.7]	0.9 [0.4–2.1]	**2.8 [1.8–4.2]**	0.8[0.5–1.3]	0.9 [0.7–1.2]	1.7 [0.9–3.2]
*Cryptosporidium* spp.	44	0.8 [0.6–1.1]****	–	–	–	–	–
**VIRAL** **PATHOGEN**	1149	22.1 [20.8–23.4]	40.5 [34.6–46.6]	**0.5 [0.4–0.6]**	**0.7 [0.6–0.8]**	**0.7 [0.7–0.8]**	**1.9 [1.6–2.3]**
Norovirus	496	9.5 [8.7–10.3]	9.5 [6.3–14.1]	**0.4 [0.2–0.5]**	0.8 [0.7–1.0]	**0.6 [0.5–0.7]**	**2.0 [1.5–2.7]**
Sapovirus	203	3.9 [3.4–4.4]	2.2 [1.1–4.5]	0.7 [0.4–1.1]	0.8 [0.6–1.4]	1.1 [0.9–1.5]	1.4 [0.8–2.5]
Rotavirus	171	3.3 [2.8–3.8]	0.5 [0.1–1.5]	**0.5 [0.3–0.9]**	**0.3 [0.2–0.5]**	**0.7 [0.5–0.9]**	**2.5 [1.5–3.9]**
Astrovirus	144	2.8 [2.3–3.2]	0.4 [0.1–1.9]	0.7 [0.4–1.2]	**0.2 [0.1–0.3]**	0.8 [0.6–1.1]	1.7 [0.9–3.2]
Adenovirus	135	2.7 [2.3–3.2]	1.7 [0.8–3.8]	**0.5 [0.3–0.9]**	**0.1 [0.1–0.3]**	**0.4 [0.3–0.6]**	1.3 [0.7–2.7]

*The total number of fecal samples positive for a specific enteropathogen divided by the total number of fecal samples analyzed.

**Prevalence estimates are based on a 0–2 year old child with no gastrointestinal symptoms sampled during the winter in the year 2011/2012 using pathogen-specific multilevel mixed-effects (MME) logistic regression models. These models were fitted with two random-effects, one at the level of the DCC and one at the level of the individual child, and were used to estimate the associations between the age, season, year and gastrointestinal symptoms at time of sampling and the presence of the enteropathogen under study. Using the fitted MME logistic regression models, we estimated the enteropathogen prevalence in the feces of an asymptomatic child aged of 0–2 years old during the winter season in 2011/2012.

***Significant odds ratios (ORs) are indicated in boldface.

****Given the small number of detections the crude, rather than the estimated, prevalence, is given.

**Figure 1:**
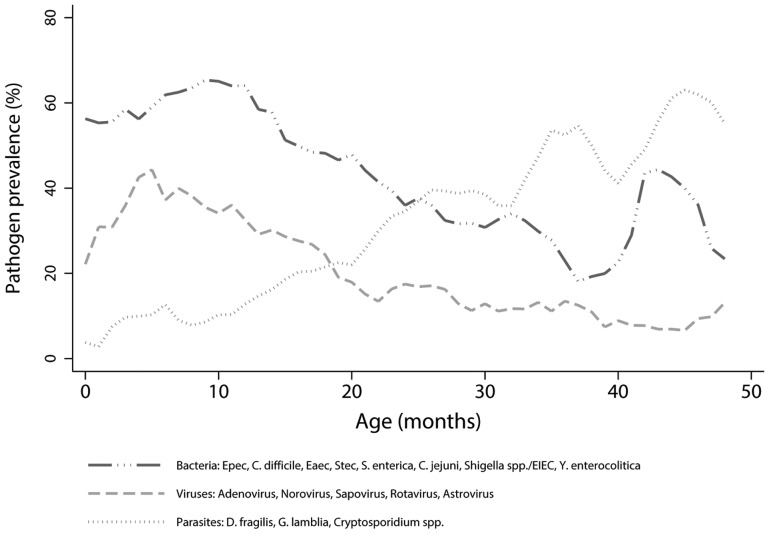
5-weekly smoothed prevalence (%) of enteropathogenic bacteria, viruses and parasites per age group in months.

**Figure 2:**
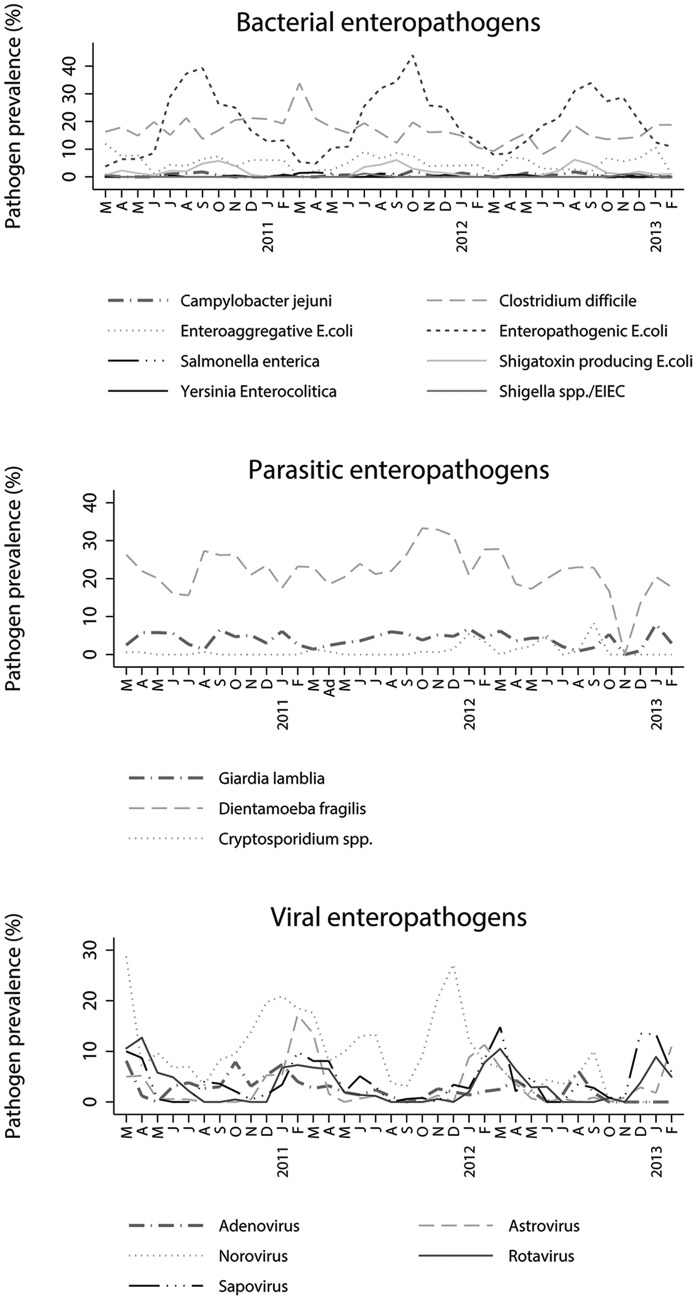
5-weekly smoothed prevalence (%) of enteropathogens of bacterial (2A), parasitic (2B) and viral (2C) origin measured during the study period.

Significant associations were found between pathogen prevalence and the child’s age. The probability of detecting any viral enteropathogen decreased with increasing age of the sampled child, while the opposite association was observed for any enteropathogenic parasite (figure 1). Compared to children 0–2 years of age, children 3–4 years of age had a lower odds of being colonized by an enteropathogen of bacterial (OR: 0.7 [0.6–0.7] or viral (OR: 0.5 [0.4–0.6]) origin, but a higher odds of being colonized by a enteropathogen of parasitic (OR: 2.3 [2.0–2.7]) origin (figure 2).

The prevalence of some enteropathogens displayed distinct seasonal patterns. (figure 2). The odds of detecting the bacterial enteropathogens EPEC and EAEC during winter months was much lower as compared to summer months (OR: 0.2 [0.2–0.3] and 0.1 [0.1–0.3] respectively). Viral enteropathogens, including rotavirus, astrovirus and adenovirus likewise displayed yearly seasonal trends, but with peak prevalence’s during the winter months. The odds of finding these enteropathogens in winter were respectively 3.7 [2.1–6.4], 6.8 [3.3–14.0] and 8.8 [4.0–20.0] times as high compared to finding them in summer and, including norovirus, were more prevalent in the reference year 2011–2012 compared to the other surveillance years. The parasitic enteropathogens showed no notable seasonal trends and were detected at relatively constant rates throughout the study period. After adjusting for the effects of the child’s age and the season and year of sampling, only the presence of norovirus (OR: 2.0 [1.5–2.7]) and rotavirus (OR: 2.5 [1.5–3.9]) was significantly and independently associated with gastroenteritis among day care attendees.

## Discussion

To our knowledge, this is the first study to estimate the prevalence of a broad range of enteropathogens in the intestinal flora of day care-attending children over a three-year time-period, its dependency on the age of the child and the season of sampling. Moreover, the study considered random factors related to the characteristics of individual child and the DCC. We demonstrated high levels and variation of detected enteropathogens among attending children and between seasons and years. Our study indicates that asymptomatic infections with enteropathogens in day care attendees are not a rare event and that gastroenteritis caused by infections with these enteropathogens is only one expression of their presence. Furthermore, we demonstrate that the gastrointestinal disease burden in day care is primarily caused by rotavirus and norovirus as these pathogens were significantly associated with gastrointestinal symptoms among attendees.

The high asymptomatic prevalence of enteropathogens in the intestine of young children, and the major role of rotavirus and norovirus in gastroenteritis, are in general agreement with other studies performed in the general population [17], the hospital [2] and the general practice setting in the Netherlands [18]. These studies however did report significantly lower prevalence estimates for *D. fragilis.* The differences in reported prevalence of *D. fragilis* between these studies and our study are likely to be related to several factors, including the different age distributions of, and antibiotic prescription rates in, the child populations studied as well as the laboratory techniques applied. In [2], 80% of children was younger than 2 years of age, versus 55% in our study. The probability of detecting *D. fragilis* is higher in a child aged 2–4 years compared to a child aged 0–2 years old, as we observed. Although seldom indicated for diarrhoea, antibiotics are more likely to have been prescribed to children in [2] and [18] for the treatment of severe GE compared to asymptomatic day care attendees on which we based our prevalence estimates. These antibiotic treatments may affect the microbiotia composition of a child’s intestine, potentially lowering the prevalence of *D. fragilis* [19]. Finally, [17] and [18] used microscopic examination to detect enteropathogens of parasitic origin, which may have been less sensitive in detecting *D. fragilis* compared to the mPCR techniques used in our study [12].

Some study limitations need to be addressed. First of all, our study may have failed to detect weaker associations between enteric infections and the presence of gastroenteritis symptoms because few children with symptoms were sampled (n = 249). Naturally, symptomatic children were underrepresented in our study because of day care absence due to their illness, which is why we provided prevalence estimates only for children without gastrointestinal symptoms at time of sampling. Secondly, some children sampled in this study might have been classified as asymptomatic, while actually being pre- or post-symptomatic. Underrepresentation of gastroenteritis cases may also have occurred if day care staff considered the norm of the individual child into account when assessing gastroenteritis status. For example, some children have many bouts of diarrhoea and/or vomiting during the first years of their life [20]. Finally, although a previous study confirmed the day care center network to be representative for the Dutch day care center population in terms of socioeconomic classification, degree of urbanization, facility design, and hygiene practicesin a study published earlier [4], it might be possible that the network was less representative with respect to some DCC characteristics for which we had no information.

Although molecular diagnostics have increased our ability to detect and identify microbiological agents, they have also complicated the clinical interpretation of the positive findings in individuals showing no signs or symptoms of GE [21]–[24]. For children, asymptomatic episodes might allow them to develop a mild yet strong enough immune response, partly protecting them against future exposures involving higher enteropathogenic doses or in periods of increased vulnerability. However, in this study no combination of enteropathogens was found to be significantly more or less prevalent than expected based on their individual prevalence. For society, we cannot exclude the possibility that asymptomatic day care-attendees may transmit enteropathogens to other children, day care staff and household members. Although the probability of such transmission events are likely to be higher for symptomatic rather than asymptomatic individuals [24], a previous study showed that the viral loads of norovirus in healthy children were within disease-causing range [25]. Furthermore, high secondary attack rates by *Shigella* spp., *G. lamblia*, rotavirus and norovirus from children to household members have been described [26]–[28], leading to increased work absenteeism and health care costs [29]. Elucidating the true clinical relevance and public health ramifications of the high prevalence of enteropathogens in asymptomatic day care attendees will remain an area of study and debate in the future.

Our next analyzes will focus on assessing the risk factors of day-care associated gastroenteritis and the societal cost of illness. Together with the prevalence estimates presented here, such analyses will facilitate the rapid and comprehensive assessment of the future impact of gastroenteritis trends in the day care setting. The role of asymptomatic and mixed infections into the etiology of disease might also prove to be a fruitful area for further study.

## References

[1] Guerrant RL, Hughes JM, Lima NL, Crane J (1990) Diarrhea in developed and developing countries: magnitude, special settings, and etiologies. Reviews of infectious diseases 12 Suppl 1S41–50. 2406855 10.1093/clinids/12.Supplement_1.S41 PMC7792920

[2] Friesema IHM, de Boer RF, Duizer E, Kortbeek LM, Notermans DW, et al (2012) Etiology of acute gastroenteritis in children requiring hospitalization in the Netherlands. European journal of clinical microbiology & infectious diseases : official publication of the European Society of Clinical Microbiology 31: 405–415. 10.1007/s10096-011-1320-0 21725865

[3] Doorduyn Y, Van Pelt W, Havelaar AH (2012) The burden of infectious intestinal disease (IID) in the community: a survey of self-reported IID in The Netherlands. Epidemiology and infection 140: 1185–1192. 21943704 10.1017/S0950268811001099

[4] Enserink R, Ypma R, Donker GA, Smit HA, van Pelt W (2013) Infectious disease burden related to child day care in the Netherlands. The Pediatric infectious disease journal 32: e334–340. 23584578 10.1097/INF.0b013e318290601e

[5] Grimprel E, Garbarg-Chenon A, Pirçon J-Y, Curran D, Soriano-Gabarró M, et al (2010) Surveillance to estimate the burden of rotavirus gastroenteritis in children aged less than 3 years attending day care centers in Paris, France. Human vaccines 6: 399–406. 20431346 10.4161/hv.6.5.11021

[6] Ferreira MSR, Xavier MDPTP, Tinga ACDC, Rose TL, Fumian TM, et al (2012) Assessment of gastroenteric viruses frequency in a children’s day care center in Rio De Janeiro, Brazil: a fifteen year study (1994–2008). PloS one 7: e33754–e33754. 22448271 10.1371/journal.pone.0033754 PMC3309004

[7] Hjelt K, Nielson OH, Paerregaard A, Grauballe PC, Krasilnikoff PA (1987) Acute gastroenteritis in children attending day-care centres with special reference to rotavirus infections. II. Clinical manifestations. Acta paediatrica Scandinavica 76: 763–768. 3661179 10.1111/j.1651-2227.1987.tb10562.x

[8] Lyman WH, Walsh JF, Kotch JB, Weber DJ, Gunn E, et al (2009) Prospective study of etiologic agents of acute gastroenteritis outbreaks in child care centers. The Journal of pediatrics 154: 253–257. 18783794 10.1016/j.jpeds.2008.07.057

[9] Ferguson JK, Jorm LR, Allen CD, Whitehead PK, Gilbert GL (1995) Prospective study of diarrhoeal outbreaks in child long-daycare centres in western Sydney. The Medical journal of Australia 163: 137–140. 7643764 10.5694/j.1326-5377.1995.tb127962.x

[10] Gallimore CI, Cubitt D, du Plessis N, Gray JJ (2004) Asymptomatic and symptomatic excretion of noroviruses during a hospital outbreak of gastroenteritis. Journal of clinical microbiology 42: 2271–2274. 15131210 10.1128/JCM.42.5.2271-2274.2004 PMC404621

[11] Enserink R, Noel H, Friesema I, Jager Cd, Kooistra M, et al (2012) The KIzSS network, a sentinel surveillance system for infectious diseases in day care centers: study protocol. BMC infectious diseases 12: 259–259. 23066727 10.1186/1471-2334-12-259 PMC3561242

[12] de Boer RF, Ott A, Kesztyüs B, Kooistra-Smid AMD (2010) Improved detection of five major gastrointestinal pathogens by use of a molecular screening approach. Journal of clinical microbiology 48: 4140–4146. 20861334 10.1128/JCM.01124-10 PMC3020836

[13] de Boer RF, Wijma JJ, Schuurman T, Moedt J, Dijk-Alberts BG, et al (2010) Evaluation of a rapid molecular screening approach for the detection of toxigenic Clostridium difficile in general and subsequent identification of the tcdC Δ117 mutation in human stools. Journal of microbiological methods 83: 59–65. 20674616 10.1016/j.mimet.2010.07.017

[14] Verweij JJ, Mulder B, Poell B, van Middelkoop D, Brienen EaT, et al (2007) Real-time PCR for the detection of Dientamoeba fragilis in fecal samples. Molecular and cellular probes 21: 400–404. 17587544 10.1016/j.mcp.2007.05.006

[15] Verweij JJ, Blangé RA, Templeton K, Schinkel J, Brienen EAT, et al (2004) Simultaneous detection of Entamoeba histolytica, Giardia lamblia, and Cryptosporidium parvum in fecal samples by using multiplex real-time PCR. Journal of clinical microbiology 42: 1220–1223. 15004079 10.1128/JCM.42.3.1220-1223.2004 PMC356880

[16] Svraka S, van der Veer B, Duizer E, Dekkers J, Koopmans M, et al (2009) Novel approach for detection of enteric viruses to enable syndrome surveillance of acute viral gastroenteritis. Journal of clinical microbiology 47: 1674–1679. 19339472 10.1128/JCM.00307-09 PMC2691081

[17] de Wit MA, Koopmans MP, Kortbeek LM, Wannet WJ, Vinjé J, et al (2001) Sensor, a population-based cohort study on gastroenteritis in the Netherlands: incidence and etiology. American journal of epidemiology 154: 666–674. 11581101 10.1093/aje/154.7.666

[18] de Wit MA, Koopmans MP, Kortbeek LM, van Leeuwen NJ, Vinjé J, et al (2001) Etiology of gastroenteritis in sentinel general practices in the netherlands. Clinical infectious diseases : an official publication of the Infectious Diseases Society of America 33: 280–288. 11438890 10.1086/321875

[19] Nagata N, Marriott D, Harkness J, Ellis JT, Stark D (2012) Current treatment options for Dientamoeba fragilis infections. International Journal for Parasitology: Drugs and Drug Resistance 2: 204–215. 24533282 10.1016/j.ijpddr.2012.08.002 PMC3862407

[20] Huskins WC (2000) Transmission and control of infections in out-of-home child care. The Pediatric infectious disease journal 19: S106–110. 11052398 10.1097/00006454-200010001-00004

[21] Phillips G, Lopman B, Tam CC, Iturriza-Gomara M, Brown D, et al (2009) Diagnosing norovirus-associated infectious intestinal disease using viral load. BMC infectious diseases 9: 63–63. 19442278 10.1186/1471-2334-9-63 PMC2698835

[22] Carman WF, Niesters HG (2007) The end of cell culture in diagnostics: is molecular diagnosis the Harry Potter or the Lord Voldemort of clinical virology as a specialty? South African medical journal = Suid-Afrikaanse tydskrif vir geneeskunde 97: 1169–1176. 18250931

[23] Gunson RN, Miller J, Leonard A, Carman WF (2003) Importance of PCR in the diagnosis and understanding of rotavirus illness in the community. Communicable disease and public health/PHLS 6: 63–65. 12736976

[24] Sukhrie FHa, Teunis P, Vennema H, Copra C, Thijs Beersma MFC, et al (2012) Nosocomial transmission of norovirus is mainly caused by symptomatic cases. Clinical infectious diseases : an official publication of the Infectious Diseases Society of America 54: 931–937. 22291099 10.1093/cid/cir971

[25] Ayukekbong J, Lindh M, Nenonen N, Tah F, Nkuo-Akenji T, et al (2011) Enteric viruses in healthy children in Cameroon: viral load and genotyping of norovirus strains. Journal of medical virology 83: 2135–2142. 22012721 10.1002/jmv.22243

[26] Fogarty J (1996) Infectious disease risk in créche, day-care and pre-school. Irish medical journal 89: 210, 212–210, 212. 8996944

[27] Rodriguez WJ, Kim HW, Brandt CD, Yolken RH, Richard M, et al (1979) Common exposure outbreak of gastroenteritis due to type 2 rotavirus with high secondary attack rate within families. The Journal of infectious diseases 140: 353–357. 227970 10.1093/infdis/140.3.353

[28] Butz AM, Larson E, Fosarelli P, Yolken R (1990) Occurrence of infectious symptoms in children in day care homes. American journal of infection control 18: 347–353. 2285172 10.1016/0196-6553(90)90248-q

[29] Van der Wielen M, Giaquinto C, Gothefors L, Huelsse C, Huet F, et al (2010) Impact of community-acquired paediatric rotavirus gastroenteritis on family life: data from the REVEAL study. BMC family practice 11: 22–22. 20230601 10.1186/1471-2296-11-22 PMC2841655

